# Bis{2-[(pyridin-4-yl-κ*N*)sulfan­yl]pyrazine}­silver(I) tetra­fluoridoborate

**DOI:** 10.1107/S1600536811051270

**Published:** 2011-12-03

**Authors:** Zi-Jia Wang

**Affiliations:** a Department of Chemistry, Capital Normal University, Beijing 100048, People’s Republic of China

## Abstract

In the title mononuclear complex, [Ag(C_9_H_7_N_3_S)_2_]BF_4_, the Ag^I^ ion adopts a virtually linear coordination geometry [N—Ag—N = 178.06 (11)°] with the two ligands bound to the metal atom *via* the pyridine N atoms. The metal-coordinated pyridine rings are almost coplanar, making a dihedral angle of 1.5 (2)°, while the two pendent pyrazine rings are arranged on the same side of the N—Ag—N line. Along the *a* axis, the mononuclear coordination units are stacked with π–π inter­actions between the pyridine rings [centroid–centroid distance = 3.569 (4) Å], leading to infinite chains. The chains are inter­connected through inter­molecular N(pyrazine)⋯π(pyrazine) inter­actions forming layers parallel to the *ab* plane [N⋯centroid = 3.268 (5) Å]. These layers are further stacked along the *c*-axis direction, furnishing a three-dimensional supra­molecular framework with the tetra­fluoridoborate anions embedded within the inter­stices.

## Related literature

For metal complexes with chalcogenobispyridines and derivates, see: Baradello *et al.* (2004 ▶); Dunne *et al.* (1997 ▶). For the crystal structures of di-2-pyridyl sulfide and its *N*-positional isomer complexes, see: Jung *et al.* (2001 ▶, 2003 ▶). For the N(pyrazin­yl)⋯centroid(pyrazin­yl) distance in {[Ni(*L*)(NO_3_)_2_]}_∞_ (*L* = bis­(2-pyrazylmeth­yl)sulfide), see: Black *et al.* (2007 ▶); For van der Waals radii, see: Bondi (1964 ▶) and for the half thickness of phenyl rings, see: Malone *et al.* (1997 ▶).
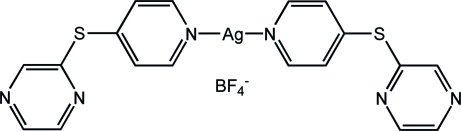


## Experimental

### 

#### Crystal data

[Ag(C_9_H_7_N_3_S)_2_]BF_4_
*M* *_r_* = 573.15
Monoclinic, 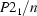

*a* = 7.2232 (2) Å
*b* = 16.4826 (3) Å
*c* = 17.6098 (4) Å
β = 91.666 (1)°
*V* = 2095.69 (8) Å^3^
*Z* = 4
Mo *K*α radiation
μ = 1.22 mm^−1^
*T* = 296 K
0.40 × 0.30 × 0.20 mm

#### Data collection

Bruker SMART APEXII CCD area-detector’ diffractometer
Absorption correction: multi-scan (*SADABS*; Bruker, 2007 ▶) *T*_min_ = 0.616, *T*_max_ = 0.746
14794 measured reflections
3597 independent reflections
3215 reflections with *I* > 2σ(*I*)
*R*_int_ = 0.028

#### Refinement

*R*[*F* ^2^ > 2σ(*F* ^2^)] = 0.036
*wR*(*F* ^2^) = 0.116
*S* = 1.11
3597 reflections
289 parameters
H-atom parameters constrained
Δρ_max_ = 0.40 e Å^−3^
Δρ_min_ = −0.62 e Å^−3^

### 

Data collection: *APEX2* (Bruker, 2007 ▶); cell refinement: *SAINT* (Bruker, 2007 ▶); data reduction: *SAINT*; program(s) used to solve structure: *SHELXS97* (Sheldrick, 2008 ▶); program(s) used to refine structure: *SHELXL97* (Sheldrick, 2008 ▶); molecular graphics: *SHELXTL* (Sheldrick, 2008 ▶); software used to prepare material for publication: *SHELXTL* and *PLATON* (Spek, 2009 ▶).

## Supplementary Material

Crystal structure: contains datablock(s) I, global. DOI: 10.1107/S1600536811051270/zq2141sup1.cif

Structure factors: contains datablock(s) I. DOI: 10.1107/S1600536811051270/zq2141Isup2.hkl

Additional supplementary materials:  crystallographic information; 3D view; checkCIF report

## Figures and Tables

**Fig. 1.**
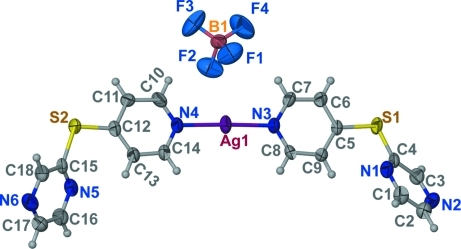
The atom-numbering scheme of the title complex. Displacement ellipsoids are drawn at the 50% probability level, while the H atoms are shown as rods of arbitrary radius.

**Fig. 2.**
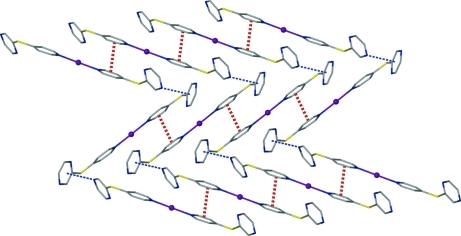
The π–π stacking and N···π(pyrazinyl) interactions between the mononuclear units, which are respectively shown as thick red-dashed lines and thin blue-dashed lines.

**Fig. 3.**
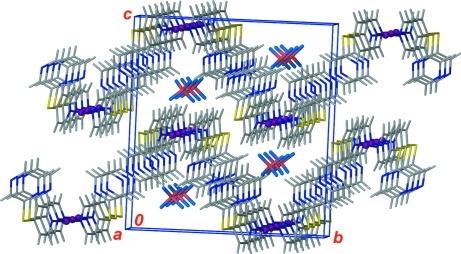
The packing structure of the title complex. The AgI ions are shown as purple balls, while the B-F bonds are shown as thick bond mode for clarity.

## supplementary crystallographic information

### Comment

Chalcogenobispyridines and derivates were widely used as versatile building blocks for supramolecular assembly (Baradello *et al.*, 2004; Dunne *et al.*, 1997). The ligand, such as the di-2-pyridyl sulfide and its N-positional isomers, endowed with the rotatable C(*sp*^2^)—S bond and a variable C(*sp*^2^)—S—C(*sp*^2^) angle (about 100°), exhibits flexible ligation modes in construction of diverse coordination motifs with unusual properties (Jung *et al.*, 2001; Jung *et al.*, 2003). Herein, we report a new silver complex derived from the 2-(pyridin-4-ylsulfanyl)pyrazine ligand.

In the mononuclear complex, [Ag(C_9_H_7_N_3_S)_2_]^+^.BF_4_^-^, the siver(I) ion adopts a linear coordination geometry [N3—Ag1—N4 = 178.06 (1)°] with the two ligands bound to the metal center via the 4-pyridyl N atoms (Fig. 1). The two pyridyl rings bound to Ag^I^ are almost coplanar, while the two pendent pyrazinyl rings are arranged on the same side of the N—Ag—N line. The dihedral angle between the mean planes of the two pendent pyrazinyl rings is 48.89 (1)°. Along the *a* axis, the mononuclear units are stacked with π–π interactions between the 4-pyridyl rings [Cg1···Cg2^i^ 3.569 (4) Å; symmetry code: (i) = x+1, y, z] leading to infinite chains (Cg1 = C5-C6-C7-N3-C8-C9; Cg2 = N4-C10-C11-C12-C13-C14). The formed chains interconnect through intermolecular N(pyrazinyl)···π(pyrazinyl) interactions forming layers parallel to the *ab* plane (Fig. 2). For the N(pyrazinyl)···π(pyrazinyl) contact, the N6···Cg3^ii^ distance equals 3.268 (5) Å (Cg3 = N1-C1-C2-N2-C3-C4; (ii) = -x, -y, -z) which is comparable to that N(pyrazinyl)···centroid(pyrazinyl) of 3.05 Å reported by Black *et al.* (2007) in {[Ni(L)(NO_3_)_2_]}_∞_ (L = bis(2-pyrazylmethyl)sulfide). These distances are shorter than the van der Waals separation of 3.40 Å on the basis of Pauling's values for the half thickness of phenyl rings (1.85 Å) (Malone *et al.*, 1997) and the van der Waals radius of N (1.55 Å) (Bondi, 1964). The almost parallel layers are further stacked along the *c* direction to furnish a three-dimensional supramolecular framework with the tetrafluoridoborate anions embedded within the interstices (Fig. 3).

### Experimental

4-Pyridyl-2-pyrazinyl sulfide was synthesized by reacting 2-chloropyrazine (0.6 g, 5.2 mmol) with sodium pyridine-4-thiolate (5 mmol) in 40 ml methanol. The mixture was refluxed with stirring for 10 hours under the protection of N2. After filtration and concentration in vacuo, the obtained crude product was further purified by chromatography on silica gel using ether acetate/dichloromethane (1:3) as the eluent, giving 0.444 g of yellow powder of 4-pyridyl-2-pyrazinyl sulfide in 47% yield. Reaction of 4-pyridyl-2-pyrazinyl sulfide (19 mg, 0.1 mmol) and AgBF_4_ (20 mg, 0.1 mmol) in 4 ml methanol with stirring at room temperature for 3 hours. The obtained clear solution was filtrated, and the filtration was left evaporation in air. After about one week, the block-like crystals of the title complex were deposited (18.1 mg, yield 63%).

### Refinement

The H atoms were placed in idealized positions and allowed to ride on the relevant carbon atoms, with C—H = 0.93 Å and *U_iso_*(H) = 1.2*U_eq_*(C).

### Crystal data

**Table:**

[Ag(C_9_H_7_N_3_S)_2_]BF_4_	*Z* = 4
*M**_r_* = 573.15	*F*(000) = 1136
Monoclinic, *P*2_1_/*n*	*D*_x_ = 1.817 Mg m^−^^3^
Hall symbol: -P 2yn	Mo *K*α radiation, λ = 0.71073 Å
*a* = 7.2232 (2) Å	µ = 1.22 mm^−^^1^
*b* = 16.4826 (3) Å	*T* = 296 K
*c* = 17.6098 (4) Å	Block, yellow
β = 91.666 (1)°	0.40 × 0.30 × 0.20 mm
*V* = 2095.69 (8) Å^3^	

### Data collection

**Table:**

'Bruker SMART APEXII CCD area-detector' diffractometer	3597 independent reflections
Radiation source: fine-focus sealed tube	3215 reflections with *I* > 2σ(*I*)
graphite	*R*_int_ = 0.028
ω scans	θ_max_ = 25.0°, θ_min_ = 2.3°
Absorption correction: multi-scan (*SADABS*; Bruker, 2007)	*h* = −8→8
*T*_min_ = 0.616, *T*_max_ = 0.746	*k* = −19→19
14794 measured reflections	*l* = −20→20

### Refinement

**Table:**

Refinement on *F*^2^	Primary atom site location: structure-invariant direct methods
Least-squares matrix: full	Secondary atom site location: difference Fourier map
*R*[*F*^2^ > 2σ(*F*^2^)] = 0.036	Hydrogen site location: inferred from neighbouring sites
*wR*(*F*^2^) = 0.116	H-atom parameters constrained
*S* = 1.11	*w* = 1/[σ^2^(*F*_o_^2^) + (0.0584*P*)^2^ + 2.8465*P*] *P* = (*F*_o_^2^ + 2*F*_c_^2^)/3
3597 reflections	(Δ/σ)_max_ = 0.001
289 parameters	Δρ_max_ = 0.40 e Å^−^^3^
0 restraints	Δρ_min_ = −0.62 e Å^−^^3^

### Special details

**Table:**

Geometry. All esds (except the esd in the dihedral angle between two l.s. planes) are estimated using the full covariance matrix. The cell esds are taken into account individually in the estimation of esds in distances, angles and torsion angles; correlations between esds in cell parameters are only used when they are defined by crystal symmetry. An approximate (isotropic) treatment of cell esds is used for estimating esds involving l.s. planes.
Refinement. Refinement of F^2^ against ALL reflections. The weighted R-factor wR and goodness of fit S are based on F^2^, conventional R-factors R are based on F, with F set to zero for negative F^2^. The threshold expression of F^2^ > 2sigma(F^2^) is used only for calculating R-factors(gt) etc. and is not relevant to the choice of reflections for refinement. R-factors based on F^2^ are statistically about twice as large as those based on F, and R- factors based on ALL data will be even larger.

### Fractional atomic coordinates and isotropic or equivalent isotropic displacement parameters (Å^2^)

**Table:**

	*x*	*y*	*z*	*U*_iso_*/*U*_eq_	
Ag1	0.49553 (4)	0.746244 (18)	0.040102 (19)	0.04882 (15)	
S1	1.31192 (14)	0.55322 (8)	0.04143 (6)	0.0556 (3)	
S2	−0.31897 (14)	0.94030 (8)	0.04687 (6)	0.0554 (3)	
N1	1.3844 (4)	0.62555 (19)	0.1758 (2)	0.0456 (8)	
N2	1.5853 (5)	0.4934 (2)	0.2314 (2)	0.0557 (9)	
N3	0.7552 (4)	0.68164 (18)	0.04124 (17)	0.0392 (7)	
N4	0.2344 (4)	0.81040 (18)	0.04305 (18)	0.0409 (7)	
N5	−0.3990 (4)	0.85798 (19)	0.1733 (2)	0.0459 (8)	
N6	−0.5935 (5)	0.9876 (2)	0.2337 (2)	0.0582 (10)	
C1	1.4578 (6)	0.6243 (3)	0.2450 (3)	0.0532 (10)	
H1A	1.4393	0.6685	0.2767	0.064*	
C2	1.5609 (6)	0.5599 (3)	0.2723 (3)	0.0576 (11)	
H2A	1.6150	0.5630	0.3208	0.069*	
C3	1.5056 (6)	0.4919 (2)	0.1643 (2)	0.0482 (9)	
H3A	1.5164	0.4456	0.1346	0.058*	
C4	1.4040 (5)	0.5577 (2)	0.1353 (2)	0.0387 (8)	
C5	1.0957 (5)	0.6030 (2)	0.0462 (2)	0.0378 (8)	
C6	1.0216 (6)	0.6326 (3)	−0.0213 (2)	0.0499 (10)	
H6A	1.0862	0.6276	−0.0660	0.060*	
C7	0.8514 (6)	0.6697 (3)	−0.0217 (2)	0.0487 (10)	
H7A	0.8005	0.6874	−0.0679	0.058*	
C8	0.8306 (5)	0.6540 (3)	0.1058 (2)	0.0458 (9)	
H8A	0.7667	0.6626	0.1502	0.055*	
C9	0.9972 (5)	0.6135 (3)	0.1114 (2)	0.0479 (10)	
H9A	1.0421	0.5938	0.1579	0.057*	
C10	0.1515 (6)	0.8409 (3)	−0.0177 (3)	0.0569 (11)	
H10A	0.2110	0.8369	−0.0637	0.068*	
C11	−0.0205 (6)	0.8788 (3)	−0.0173 (2)	0.0546 (11)	
H11A	−0.0779	0.8967	−0.0622	0.066*	
C12	−0.1045 (5)	0.8893 (2)	0.0520 (2)	0.0398 (8)	
C13	−0.0160 (5)	0.8602 (3)	0.1160 (2)	0.0472 (9)	
H13A	−0.0676	0.8671	0.1634	0.057*	
C14	0.1513 (5)	0.8204 (3)	0.1094 (2)	0.0462 (9)	
H14A	0.2089	0.7997	0.1532	0.055*	
C15	−0.4147 (5)	0.9284 (2)	0.1376 (2)	0.0373 (8)	
C16	−0.4753 (6)	0.8546 (3)	0.2415 (3)	0.0560 (11)	
H16A	−0.4609	0.8077	0.2703	0.067*	
C17	−0.5735 (6)	0.9177 (3)	0.2701 (3)	0.0580 (11)	
H17A	−0.6283	0.9115	0.3169	0.070*	
C18	−0.5097 (6)	0.9934 (2)	0.1678 (2)	0.0457 (9)	
H18A	−0.5148	1.0422	0.1412	0.055*	
B1	0.4920 (7)	0.7584 (3)	−0.1658 (3)	0.0479 (11)	
F1	0.5771 (6)	0.8120 (2)	−0.1155 (2)	0.0970 (11)	
F2	0.4070 (6)	0.7015 (2)	−0.1210 (2)	0.0977 (11)	
F3	0.3676 (6)	0.7987 (3)	−0.2082 (3)	0.1304 (16)	
F4	0.6212 (6)	0.7205 (3)	−0.2054 (3)	0.1233 (15)	

### Atomic displacement parameters (Å^2^)

**Table:**

	*U*^11^	*U*^22^	*U*^33^	*U*^12^	*U*^13^	*U*^23^
Ag1	0.0314 (2)	0.0524 (2)	0.0627 (2)	0.01446 (12)	0.00139 (14)	−0.00343 (13)
S1	0.0364 (5)	0.0807 (8)	0.0494 (6)	0.0263 (5)	−0.0019 (4)	−0.0044 (5)
S2	0.0389 (5)	0.0792 (8)	0.0483 (6)	0.0271 (5)	0.0076 (4)	0.0090 (5)
N1	0.0355 (17)	0.0401 (17)	0.061 (2)	0.0034 (13)	0.0009 (15)	0.0044 (15)
N2	0.053 (2)	0.057 (2)	0.056 (2)	0.0147 (17)	−0.0056 (17)	0.0105 (17)
N3	0.0299 (15)	0.0427 (17)	0.0450 (17)	0.0059 (12)	−0.0009 (13)	−0.0034 (13)
N4	0.0309 (15)	0.0422 (17)	0.0496 (18)	0.0059 (12)	0.0039 (13)	0.0010 (13)
N5	0.0373 (17)	0.0398 (17)	0.061 (2)	0.0029 (13)	0.0068 (15)	−0.0034 (15)
N6	0.057 (2)	0.061 (2)	0.057 (2)	0.0122 (17)	0.0154 (18)	−0.0127 (18)
C1	0.045 (2)	0.050 (2)	0.064 (3)	0.0002 (18)	0.001 (2)	−0.0068 (19)
C2	0.048 (2)	0.071 (3)	0.054 (3)	0.004 (2)	−0.0018 (19)	0.004 (2)
C3	0.046 (2)	0.042 (2)	0.057 (2)	0.0077 (17)	0.0040 (18)	0.0045 (18)
C4	0.0239 (16)	0.042 (2)	0.051 (2)	0.0038 (14)	0.0030 (15)	0.0082 (16)
C5	0.0277 (17)	0.0403 (19)	0.045 (2)	0.0046 (14)	−0.0009 (14)	−0.0010 (15)
C6	0.042 (2)	0.068 (3)	0.040 (2)	0.0146 (19)	0.0011 (16)	−0.0026 (18)
C7	0.040 (2)	0.065 (3)	0.041 (2)	0.0171 (18)	−0.0053 (16)	0.0010 (18)
C8	0.0258 (17)	0.067 (3)	0.045 (2)	0.0037 (17)	0.0081 (15)	0.0042 (18)
C9	0.0332 (19)	0.067 (3)	0.044 (2)	0.0065 (17)	0.0023 (16)	0.0182 (18)
C10	0.052 (2)	0.066 (3)	0.054 (2)	0.023 (2)	0.023 (2)	0.010 (2)
C11	0.051 (2)	0.072 (3)	0.041 (2)	0.026 (2)	0.0096 (18)	0.015 (2)
C12	0.0314 (18)	0.0379 (19)	0.050 (2)	0.0032 (14)	0.0055 (15)	−0.0012 (15)
C13	0.0333 (19)	0.067 (3)	0.041 (2)	0.0074 (18)	0.0025 (15)	−0.0054 (18)
C14	0.0289 (18)	0.064 (2)	0.046 (2)	0.0058 (17)	−0.0004 (15)	−0.0009 (18)
C15	0.0248 (16)	0.0421 (19)	0.0450 (19)	0.0033 (14)	0.0010 (14)	−0.0050 (15)
C16	0.048 (2)	0.053 (3)	0.067 (3)	−0.0004 (19)	0.008 (2)	0.014 (2)
C17	0.052 (3)	0.075 (3)	0.048 (2)	0.009 (2)	0.0100 (19)	0.002 (2)
C18	0.042 (2)	0.042 (2)	0.054 (2)	0.0072 (16)	0.0028 (17)	−0.0039 (17)
B1	0.055 (3)	0.053 (3)	0.036 (2)	0.010 (2)	0.002 (2)	−0.0037 (18)
F1	0.132 (3)	0.073 (2)	0.085 (2)	−0.0111 (19)	−0.030 (2)	−0.0048 (16)
F2	0.129 (3)	0.073 (2)	0.093 (2)	−0.013 (2)	0.034 (2)	0.0027 (17)
F3	0.127 (4)	0.134 (4)	0.127 (4)	0.035 (3)	−0.057 (3)	0.029 (3)
F4	0.124 (3)	0.130 (3)	0.119 (3)	0.032 (3)	0.061 (3)	−0.021 (3)

### Geometric parameters (Å, °)

**Table:**

Ag1—N3	2.156 (3)	C5—C9	1.380 (5)
Ag1—N4	2.165 (3)	C6—C7	1.373 (6)
S1—C4	1.765 (4)	C6—H6A	0.9300
S1—C5	1.768 (3)	C7—H7A	0.9300
S2—C12	1.763 (4)	C8—C9	1.378 (5)
S2—C15	1.770 (4)	C8—H8A	0.9300
N1—C1	1.315 (6)	C9—H9A	0.9300
N1—C4	1.337 (5)	C10—C11	1.391 (6)
N2—C3	1.300 (6)	C10—H10A	0.9300
N2—C2	1.326 (6)	C11—C12	1.389 (5)
N3—C8	1.327 (5)	C11—H11A	0.9300
N3—C7	1.340 (5)	C12—C13	1.366 (6)
N4—C10	1.311 (5)	C13—C14	1.383 (5)
N4—C14	1.339 (5)	C13—H13A	0.9300
N5—C15	1.324 (5)	C14—H14A	0.9300
N5—C16	1.336 (6)	C15—C18	1.386 (5)
N6—C17	1.324 (6)	C16—C17	1.364 (6)
N6—C18	1.328 (5)	C16—H16A	0.9300
C1—C2	1.376 (6)	C17—H17A	0.9300
C1—H1A	0.9300	C18—H18A	0.9300
C2—H2A	0.9300	B1—F3	1.330 (6)
C3—C4	1.397 (5)	B1—F4	1.337 (6)
C3—H3A	0.9300	B1—F2	1.380 (6)
C5—C6	1.378 (5)	B1—F1	1.382 (6)
			
N3—Ag1—N4	178.06 (11)	C8—C9—C5	118.1 (4)
C4—S1—C5	104.22 (17)	C8—C9—H9A	120.9
C12—S2—C15	105.47 (18)	C5—C9—H9A	120.9
C1—N1—C4	115.8 (3)	N4—C10—C11	123.6 (4)
C3—N2—C2	116.6 (4)	N4—C10—H10A	118.2
C8—N3—C7	116.7 (3)	C11—C10—H10A	118.2
C8—N3—Ag1	120.8 (2)	C12—C11—C10	118.3 (4)
C7—N3—Ag1	122.5 (3)	C12—C11—H11A	120.9
C10—N4—C14	117.4 (3)	C10—C11—H11A	120.9
C10—N4—Ag1	123.0 (3)	C13—C12—C11	118.4 (3)
C14—N4—Ag1	119.6 (3)	C13—C12—S2	126.8 (3)
C15—N5—C16	115.5 (3)	C11—C12—S2	114.8 (3)
C17—N6—C18	116.1 (4)	C12—C13—C14	119.1 (4)
N1—C1—C2	122.4 (4)	C12—C13—H13A	120.5
N1—C1—H1A	118.8	C14—C13—H13A	120.5
C2—C1—H1A	118.8	N4—C14—C13	123.1 (4)
N2—C2—C1	121.8 (4)	N4—C14—H14A	118.4
N2—C2—H2A	119.1	C13—C14—H14A	118.4
C1—C2—H2A	119.1	N5—C15—C18	122.2 (3)
N2—C3—C4	122.1 (4)	N5—C15—S2	119.7 (3)
N2—C3—H3A	118.9	C18—C15—S2	118.1 (3)
C4—C3—H3A	118.9	N5—C16—C17	122.2 (4)
N1—C4—C3	121.1 (4)	N5—C16—H16A	118.9
N1—C4—S1	119.5 (3)	C17—C16—H16A	118.9
C3—C4—S1	119.3 (3)	N6—C17—C16	122.3 (4)
C6—C5—C9	118.4 (3)	N6—C17—H17A	118.8
C6—C5—S1	116.4 (3)	C16—C17—H17A	118.8
C9—C5—S1	125.1 (3)	N6—C18—C15	121.5 (4)
C7—C6—C5	119.2 (4)	N6—C18—H18A	119.3
C7—C6—H6A	120.4	C15—C18—H18A	119.3
C5—C6—H6A	120.4	F3—B1—F4	114.3 (5)
N3—C7—C6	123.1 (4)	F3—B1—F2	110.8 (5)
N3—C7—H7A	118.4	F4—B1—F2	108.0 (4)
C6—C7—H7A	118.4	F3—B1—F1	108.7 (4)
N3—C8—C9	124.3 (3)	F4—B1—F1	109.2 (5)
N3—C8—H8A	117.8	F2—B1—F1	105.4 (4)
C9—C8—H8A	117.8		
			
C4—N1—C1—C2	4.9 (6)	C14—N4—C10—C11	−3.5 (7)
C3—N2—C2—C1	−0.5 (7)	Ag1—N4—C10—C11	176.8 (4)
N1—C1—C2—N2	−3.1 (7)	N4—C10—C11—C12	3.9 (8)
C2—N2—C3—C4	2.0 (6)	C10—C11—C12—C13	−1.4 (7)
C1—N1—C4—C3	−3.4 (5)	C10—C11—C12—S2	178.4 (4)
C1—N1—C4—S1	−179.6 (3)	C15—S2—C12—C13	−11.0 (4)
N2—C3—C4—N1	−0.1 (6)	C15—S2—C12—C11	169.1 (3)
N2—C3—C4—S1	176.1 (3)	C11—C12—C13—C14	−1.1 (6)
C5—S1—C4—N1	−40.5 (3)	S2—C12—C13—C14	179.1 (3)
C5—S1—C4—C3	143.3 (3)	C10—N4—C14—C13	0.6 (6)
C4—S1—C5—C6	158.8 (3)	Ag1—N4—C14—C13	−179.6 (3)
C4—S1—C5—C9	−21.7 (4)	C12—C13—C14—N4	1.6 (7)
C9—C5—C6—C7	−1.5 (7)	C16—N5—C15—C18	−2.2 (6)
S1—C5—C6—C7	178.1 (3)	C16—N5—C15—S2	180.0 (3)
C8—N3—C7—C6	−1.4 (6)	C12—S2—C15—N5	−40.4 (3)
Ag1—N3—C7—C6	176.3 (3)	C12—S2—C15—C18	141.7 (3)
C5—C6—C7—N3	2.7 (7)	C15—N5—C16—C17	4.3 (6)
C7—N3—C8—C9	−1.1 (6)	C18—N6—C17—C16	−1.0 (7)
Ag1—N3—C8—C9	−178.8 (3)	N5—C16—C17—N6	−2.8 (8)
N3—C8—C9—C5	2.1 (7)	C17—N6—C18—C15	3.0 (7)
C6—C5—C9—C8	−0.7 (6)	N5—C15—C18—N6	−1.4 (6)
S1—C5—C9—C8	179.7 (3)	S2—C15—C18—N6	176.4 (3)
